# Monitoring and enforcement of Code-based legal measures to protect breastfeeding in South Asia: opportunities and bottlenecks

**DOI:** 10.3389/fpubh.2025.1412946

**Published:** 2025-06-18

**Authors:** Constance Ching, Vani Sethi, David Lawson Clark, Joo Kean Yeong, Katherine Shats, Zivai Murira, Ahmadwali Aminee, Dhammica Rowel, Golam Mohiuddin Khan, Khadheeja Ahmed, Kinley Dorji, Mazhar Iqbal, Muhammad Faisal, Phulgendra Prasad Singh, Saba Shuja

**Affiliations:** ^1^Giovine-Clark Consultancy LLC, Coxsackie, NY, United States; ^2^UNICEF Regional Office for South Asia, Kathmandu, Nepal; ^3^UNICEF Headquarters, New York, NY, United States; ^4^UNICEF Afghanistan Country Office, Kabul, Afghanistan; ^5^UNICEF Sri Lanka Country Office, Colombo, Sri Lanka; ^6^UNICEF Bangladesh Country Office, Dhaka, Bangladesh; ^7^UNICEF Maldives Country Office, Malé, Maldives; ^8^UNICEF Bhutan Country Office, Thimphu, Bhutan; ^9^UNICEF Pakistan Country Office, Islamabad, Pakistan; ^10^UNICEF Nepal Country Office, Kathmandu, Nepal

**Keywords:** international code, breastfeeding, monitoring, enforcement, South Asia, breastmilk substitutes, marketing

## Abstract

**Objective:**

To examine the bottlenecks and opportunities associated with Code monitoring and enforcement at the national level in the eight countries of South Asia region (Afghanistan, Bhutan, Bangladesh, India, Maldives, Nepal, Pakistan, and Sri Lanka).

**Design:**

Data was collected following a workshop-as-research methodology. Deductive content analysis was used to review, categorize, and analyze data. A semi-structured categorization matrix containing three main categories (background, opportunities, and bottlenecks) was developed as a guide for categorizing data on areas relevant to monitoring and enforcement.

**Findings:**

Overarching themes in bottlenecks include: (1) There is insufficient monitoring across countries, (2) Monitoring is not well-integrated into relevant enforcement mechanisms, as there is a lack of established system to efficiently ‘feed’ monitoring findings to the designated enforcement agencies, (3) Uncertainty regarding enforcement in the context of digital marketing, (4) Lack of coordination and collaboration regarding enforcement, (5) Inadequate sanctions and penalties, sometimes due to a lack of identified legal structure and adjudication system and functional administrative or enforcement mechanisms. Overarching themes in opportunities include: (1) Product registration or licensing as entry points for monitoring and enforcement, (2) authority provided in legal measures for designated agencies to carry out monitoring and enforcement actions, (3) civil society as government partners in monitoring including developing monitoring tools and strengthening systems to integrate monitoring with enforcement. Inadequately imposed.

## Highlights

- Build monitoring and enforcement activities into national routine budget.
- Impose appropriate range of (administrative and criminal) sanctions as deterrents.
- Use registration and license as key enforcement and sanction tools.
- Review or adopt new legal measures.
- Harness health system as key point for monitoring and enforcement and to prevent conflicts of interest.
- Employ newly available technologies to help detect digital violations.
- Build regional knowledge-sharing platforms for support on monitoring and enforcement.

## 1 Introduction

Almost 40% of the total global neonatal deaths take place in South Asia (33). Increasing breastfeeding practices is critical in ensuring neonatal and child survival in the region (1). Enacting and enforcing legal measures to implement the International Code of Marketing of Breast-milk Substitutes and relevant World Health Assembly (WHA) resolutions^1^ (collectively referred to as ‘the Code’) protects parents from misleading and exploitative marketing that undermines breastfeeding and optimal infant and young child feeding (2–5). Hence Code implementation is part of governments’ obligations under relevant international human rights treaties (6). The Code prohibits the promotion of breastmilk substitutes (BMS) and feeding bottles and teats, and restricts inappropriate marketing of foods for infants and young children,^2^ calling on all governments to give effect to the Code through adopting enforceable laws (2).

Seven out of the eight countries in the South Asia region^3^ have, to varying degrees, adopted the Code into national legal measures. However, some of the weak, outmoded, and inadequate laws, as well as the absence of established and/or sustained monitoring and enforcement systems to detect, investigate, prosecute and adjudicate violations of national laws continue to hinder the protection of breastfeeding (7).

### 1.1 Monitoring and enforcement

Monitoring is outlined in Article 11 of the Code, the importance of transparent and independent monitoring that is free from commercial influence as well as the necessity to scale up monitoring and enforcement while avoiding conflicts of interest have been addressed in subsequent relevant WHA resolutions (2). Monitoring is a form of systematic information-gathering which can be carried out by civil society, professional groups, organizations and individuals who are actively involved in public health, not just government officials who are vested with powers to inspect and investigate. Monitoring findings help indicate status of compliance with the Code and relevant legal measures and identify the actions needed to strengthen existing legal measures or advocate for them if none exist. Even for countries with no Code-based legal measures, monitoring provides information on inappropriate marketing practices that can be used to advocate for Code implementation. Enforcement can only take place where there are legal measures, and effective enforcement relies upon an efficient monitoring system, a range of appropriate and deterrent penalties, and a sound legal infrastructure. It is necessary for appropriate enforcement agencies to be designated with authority to carry out inspection and investigation at the national and sub-national level as necessary, and to set enforcement proceedings in motion in the event of non-compliance, culminating in the imposition of administrative sanctions or prosecution in court. Monitoring and enforcement can complement one another, as evidence gathered during monitoring can be used as evidence to prosecute companies for violations of legal measures (2).

### 1.2 Digital marketing of BMS

The fast growing digital marketing has become, over the last two decades, the predominant source of exposure to promotion of BMS and related products for parents. Digital platforms amplify the reach and power of advertising and other forms of promotion with their capacity to generate viral and bespoke marketing, making digital marketing both cost-effective and effective in increasing purchase and use of BMS and related products. In some countries, more than 80% of BMS marketing is done through digital media (8–10). Parents who experienced higher exposure to online advertisements of BMS were more likely to purchase BMS products based on the nutritional and health claims (11).

### 1.3 Digital marketing of BMS in South Asia

A scoping review on studies that documented Code violations examined 157 studies globally, and 12 of them documented Code violations on digital platforms found in the South Asia region, including Bangladesh, Bhutan, India, Maldives, Nepal, Pakistan, and Sri Lanka (8). The 12 studies from South Asia, ranging from 1998 to 2022, documented evidence of a wide range of violations including information and educational materials, digital advertisements, donations, labeling, sponsorships, and promotion through online shops. The digital platforms where violations were found included online shops and various social media platforms (see Table 1 for summary).

**Table 1:** Summary of studies that documented Code violations on digital platforms in South Asia (8).

	Study	Year	Country	Types of violations	Types of products	Digital platforms
1	Breaking the rules, stretching the rules 1998: a worldwide report on violations of the WHO/UNICEF international code of marketing of breastmilk substitutes [IBFAN (19)]	1998	Multi-country including Bangladesh	Information and educational materials Promotion to general public including digital advertisement and promotion Donations Health workers Labeling Conflicts of interest: sponsoring conferences and medical research Promotion through online shops	BMS (IF, FUF, GUMs), bottles and teats, complementary foods	Online shops
2	Breaking the rules, stretching the rules 2004: evidence of violations of the International Code of Marketing of Breastmilk Substitutes and subsequent resolutions (IBFAN (20))	2004	Multi-country including Bangladesh	Information and educational materials Promotion to general public including digital advertisement and promotion Donations Health workers Labeling Conflicts of interest: sponsoring conferences and medical research Promotion through online shops	BMS (IF, FUF, GUMs), bottles and teats, complementary foods	Social media (Facebook) and online shops
3	Assessment of corporate compliance with guidance and regulations on labels of commercially produced complementary foods sold in Cambodia, Nepal, Senegal and Tanzania [Sweet et al. (21)]	2013	Multi-country including Nepal	Labels Online shops	Complementary foods	Online shops
4	In-Country Assessments of BMS Companies’ Compliance with the International Code of Marketing of Breast-milk Substitutes 2016, Westat India Report for ATNI 2nd Charter [Durako et al. (22)]	2016	India	Overall, the media monitoring identified no television, newspaper, magazine or social media advertising. Non-compliance found in labeling, facility/store observations including on-line stores.	BMS (IF, FUF, GUMs) and other food/beverage marketed for infants <6 months or bottle feeding	Online shops
5	High consumption of commercial food products among children less than 24 months of age and product promotion in Kathmandu Valley, Nepal [Pries et al. (23)]	2016	Nepal	Twenty-eight percent of mothers reported observing a promotion for breastmilk substitutes, and 20.1% reported promotions for commercially produced complementary foods; Promotions for these same commercially produced snack food products were highly prevalent in Kathmandu Valley, reported by 85.4% of mothers.	BMS (IF, FUF, GUMs), complementary foods	Internet
6	Milking It—How Milk Formula Companies are Putting Profits Before Science 2017 (Full report and Executive Summary) [Changing Markets Foundation (24)]	2017	Multi-country including India	Promotion to general public/mothers: Advertisement and other forms of promotion Health claims Retail including online shops Cross promotion	BMS (IF and FUF)	Social media: YouTube, Instagram, Facebook
7	IBFAN-ICDC. Breaking the Rules (BTR), Stretching the Rules 2017: Evidence of violations of the International Code of Marketing of Breastmilk Substitutes and subsequent resolutions, compiled from June 2014 to June 2017	2017	India, Pakistan, Sri Lanka	Information and educational materials Promotion to general public including digital advertisement and promotion Contacting mothers through digital platforms Donations Health workers Labeling Conflicts of interest: sponsoring conferences and medical research Promotion through online shops	BMS (IF, FUF, GUMs), bottles and teats, complementary foods	Facebook, YouTube, Instagram, online shops
8	IBFAN Asia Report on the Monitoring of the Code in 11 Countries of Asia [IBFAN-ICDC (25)]	2018	Bhutan, India, Maldives, Nepal, and Sri Lanka	Health and nutritional claims have become a prime marketing tool. Many of these claims are presented as complex scientific formulations, which are then used as trademarked logos, mascots or benefit icons to create a “premium” market. Digital platforms allow companies to contact parents, collect information and carry out promotional activities in more seamless ways	BMS (IF, FUF, GUMs), bottles and teats, complementary foods	Facebook, Instagram, online shops
9	Sponsorship of national and regional professional pediatrics associations by companies that make breast-milk substitutes: evidence from a review of official websites [Grummer-Strawn et al. (26)]	2019	Africa, Americas, Asia, Europe, Oceania including Bangladesh	68 (60%) of the 114 pediatric associations with a website or Facebook account documented receiving financial support from BMS companies.	BMS (IF, FUF, GUMs)	Websites and social media: Facebook pages
10	BPNI Under Attack: A report of the monitoring the compliance with the Infant milk substitutes, Feeding bottles and Infant foods (Regulation of Production, Supply and Distribution) Act 1992 and the Amendment Act 2003. 2019-20-21	2021	India	Information and educational materials Promotion to general public including digital advertisement and promotion Contacting mothers through digital platforms Donations Health workers Labeling Conflicts of interest: sponsoring conferences and medical research	BMS (IF, FUF, GUMs) and bottles and teats	Social media: Facebook, Instagram
11	Old Tricks, New Opportunities: How Companies Violate the International Code of Marketing of Breast-Milk Substitutes and Undermine Maternal and Child Health during the COVID-19 Pandemic [Ching et al. (27)]	2021	Multi-country including India and Pakistan	Promotion to general public/mothers: Advertisement and other forms of promotion Contacting mothers through digital platforms Health claims Donations	BMS (IF, FUF, GUMs) and bottles and teats	Social media: Facebook, Instagram
12	Violations of International Code of Breast-milk Substitutes (BMS) in commercial settings and media in Bangladesh [Sheikh et al. (28)]	2022	Bangladesh	Information and educational materials Promotion to general public (mass media and retail outlets) Labeling	BMS (IF, FUF, GUMs), bottles and teats.	Online shops

### 1.4 Challenges of restricting digital marketing and the new WHO guidance on restricting digital marketing of BMS

Digital marketing produces larger amounts of materials that need to be monitored. It also involves a wider range of actors compared to traditional marketing practices. Many strategies specific to digital marketing result in precisely targeted and personalized marketing, such as virtual support groups and baby clubs, promotion disguised as selling on e-commerce platforms (online retailers), user or influencer generated content, cross-border marketing, and the use of algorithms and data mining to analyze and trade user information (10).

Definitions of promotion to general public in the Code and advertising in some national measures are logically wide enough to cover many types of marketing activities on digital platforms. However, coupled with the advances in digitalization of healthcare services, to effectively apply the Code to digital environments, in particular to enforce relevant legal measures, requires more specific and targeted regulatory mechanisms, coordination among a broader set of government agencies, and the establishment of particular legal duties on the range of entities involved in the digital marketing value chain. This includes data management platforms, content creators (including influencers), internet service providers (ISPs), social media platforms, search engine providers, online retailers, application developers and owners, gaming service providers, telemedicine patient care platforms, and appointment scheduling software (12).

Digitalization in advertising and the health systems has required some clarification of implementation mechanisms of Code provisions and relevant legal measures. These ‘new’ digital activities have created additional and grave challenges for monitoring and enforcement (10).

A study on worldwide legislation implemented to regulate BMS marketing on digital and social media reveals that, globally, only 28 documents from 24 countries include explicit provisions regarding regulations on digital and/or social media marketing. Within the South Asia region, only Bangladesh and India are found to be in this category (13).

In November 2023, WHO published the *Guidance on Regulatory Measures aimed at Restricting Digital Marketing of Breast-milk Substitutes* (the 2023 WHO Guidance), following the WHA Decision 73.26 in 2020 and WHA Decision 75.21 in 2022 that called for a review of the scope and impact of digital marketing of BMS (10) and guidance to support governments on regulatory measures (12). The 2023 Guidance aims to provide recommendations to governments specifically on developing and applying regulatory measures aimed at restricting digital marketing of products that fall within the scope of the Code and foods for infants and young children, including digital marketing in relation to humanitarian and emergency contexts. The recommendations in the 2023 WHO Guidance are summarized below in Table 2.

**Table 2:** A summary of recommendations in the guidance on regulatory measures aimed at restricting digital marketing of breast-milk substitutes (12).

	Recommendations
1.	Member States should ensure that regulatory measures effectively prohibit the promotion of products within the scope of the Code across all channels and media, including digital media.
2.	Regulatory measures should prohibit promotion of products within the scope of the Code through health care systems using digital technologies.
3.	Regulatory measures should prohibit promotion of products within the scope of the Code at point-of-sale in digital environments, in alignment with the Code provisions on point-of-sale promotions, information and education and labeling.
4.	Member States should prohibit inappropriate promotion of foods for IYC that are not BMS in digital environments.
5.	Member States should confer legal duties of compliance to monitor and take action to prevent or remedy prohibited marketing on entities along the digital marketing value chain.
6.	Regulatory measures should identify government agencies responsible for implementation, monitoring, and enforcement of the Code and the Guidance on Ending Inappropriate Promotion of Foods for IYC, including in digital environments, establish mechanisms for inter-agency collaboration, allocate adequate resources and establish powers necessary for discharging these duties.
7.	Member States should strengthen monitoring systems for capturing violations in the digital environment.
8.	Member States should enforce regulatory measures that implement the Code, including in digital environments, and apply effective, proportionate, dissuasive sanctions for non-compliance.
9.	Member States should exercise jurisdiction to ensure manufacturers and distributors of products within the scope of the Code and foods for IYC can be held liable for digital marketing practices that cross into or out of their countries and do not comply with regulatory measures that implement the Code.
10.	All entities along the digital marketing value chain and in health care systems should ensure that their marketing practices conform to the Code (including the Guidance on Ending Inappropriate Promotion of Foods for IYC) in digital environments, irrespective of any regulatory measures implemented at national and subnational levels.
11.	Member States should monitor developments in digital technologies and their impact on Code compliance and adapt regulatory measures to capture new digital technologies, channels or marketing practices.

### 1.5 Monitoring and enforcement around the globe

A global assessment by WHO and UNICEF on the status of national Code implementation in 2016 revealed that formal monitoring and enforcement mechanisms remain very limited: Out of 194 countries,^4^ only 32 reported having a mechanism in place and just six countries reported having dedicated budgets and funding for monitoring and enforcement (14). In response, WHO/UNICEF released the Network for Global Monitoring and Support for Implementation of the International Code of Marketing of Breast-milk Substitutes and Subsequent Relevant World Health Assembly Resolutions (NetCode) toolkit in 2017 to provide guidance for governments and civil society partners (15). In particular, its protocol for ongoing monitoring systems, which is designed to be integrated into existing regulatory and enforcement systems, aims to:

- detect violations of the national measures and/or the Code.
- document and report such violations.
- investigate and validate whether the reported activities are indeed violations.
- activate an enforcement mechanism that will identify interim actions for violations to immediately cease, permanently stop such violations and deter future violations; and
- hold manufacturers, distributors, retail outlets, the health-care system and health-care workers to account for their breeches of national measures.

As of 2022, only approximately 40% of 194 countries (83 countries) have identified in their legal measures the government agency or body that is responsible for monitoring compliance, and less than half (91 countries) of all countries have defined sanctions for violations (5).

### 1.6 Research gap and question

Existing research has only assessed Code implementation status in South Asia region through analyzing national Code-based legal measures (5, 7). However, there has not been any study that specifically focuses on the challenges and opportunities of Code monitoring and enforcement of national measures in the region. For instance, even though the 2022 WHO/UNICEF Code Status Report included assessments of legal provisions relating to monitoring and enforcement, the selected criteria do not sufficiently indicate how effective the monitoring or enforcement system is. Information as to whether monitoring and enforcement mechanisms are implemented or integrated into existing systems was not provided (5). Thus, this study seeks to examine specifically the bottlenecks and opportunities associated with monitoring and enforcement mechanisms at the national level in the eight countries of South Asia region (Afghanistan, Bhutan, Bangladesh, India, Maldives, Nepal, Pakistan, and Sri Lanka).

## 2 Methods

### 2.1 Workshop-as-research approach

This study, including data collection and analysis, was conducted between May and October 2023. It follows a workshop-as-research approach (16), as one of the main aims of the Regional Workshop on Strengthening Monitoring and Enforcement of Legal Measures to Protect Breastfeeding in South Asia (“the Kathmandu Workshop) was to generate data on the research topic. The workshop-as-research approach provided a platform that aids researchers in identifying and exploring relevant factors in assessing and understanding complex and multi-faceted monitoring and enforcement mechanisms and processes. This approach also supports identifying factors that are not obvious to either the participants or the researchers prior to commencing the workshop process (16). The lead author of this study, who was also one of the workshop co-facilitators, was designated as the rapporteur of the workshop to specifically document the entire workshop process. As the workshop was applied as part of the research design, the double-role of rapporteur and co-facilitator allowed for data to be documented in an immersive and collaborative environment where proactive clarification or negotiation of meaning was possible when necessary (16).

Held in Kathmandu, Nepal on 8–10 May 2023, the Kathmandu Workshop was jointly organized by the UNICEF Regional Office for South Asia (UNICEF ROSA) and WHO Regional office for South-East Asia (WHO SEARO), as a sequel to an earlier Regional Workshop held in November 2022 in Colombo, Sri Lanka which identified the lack of monitoring and enforcement as a common gap among countries in the region. The organizers consulted with the UNICEF and WHO country offices and relevant government agencies of Afghanistan, Bhutan, Bangladesh, India, Maldives, Nepal, Pakistan, and Sri Lanka on the recruitment of participants based on their role and involvement in Code monitoring and enforcement at the national level. A total of 57 participants attended the workshop. They consisted of at least two, and up to 13, representatives from each of the eight countries representing respective UNICEF and/or WHO country office and government), together with representatives from UNICEF ROSA, WHO SEARO, and UNICEF headquarters; and specialists on the International Code as trainers/facilitators.

The focus of the workshop was to identify the bottlenecks and opportunities associated with Code monitoring and enforcement of Code-related legal measures based on response and feedback gathered through the workshop, and to develop recommended actions on how to integrate monitoring into existing enforcement mechanisms and establish an ongoing and government-run monitoring system that references NetCode’s ongoing monitoring toolkit (15).

### 2.2 Data collection

Data was collected before and during the workshop, in the format of a pre-workshop questionnaire (Table 3) on different aspects of Code monitoring and enforcement of national measures which was disseminated to participants from the eight countries prior to the Kathmandu Workshop. Additionally, discussions during the workshop on the following topics were documented in a detailed report which was used as data for analysis.

- Where are we and what needs to be done?
- Familiarization with the NetCode tool kit.
- Beginning the process: what, where and when?
- Key considerations for enforcement.
- Building a national monitoring team.
- Financing of monitoring and enforcement.
- Monitoring and enforcement and developing standard monitoring tools and a database.

**Table 3:** Questions from the pre-workshop questionnaire.

	Questions from questionnaire
1.	Where is the current monitoring and enforcement system placed?
2.	If there is an existing monitoring and enforcement system, is it functioning? If not, why not?
3.	Is digital marketing being tackled? If yes, how?
4.	What have been the barriers and/or successes you have encountered in obtaining information on violations?
5.	Has there been any action taken against violators? If not, why not?
6.	Who is responsible for compliance under the law/regulations? (E.g. manufacturers, distributors, retailers, importers, advertising agencies, social media platforms, internet service providers, healthcare facilities (public and private?), health professionals, NGOs, etc.?
7.	Are there separate penalties for different types of violations? Are they adequate?
8.	Is the current system satisfactory? Are there gaps? Are capacity and resources adequate?
9.	Do relevant authorities have any interaction with industry over their marketing practices? If yes, please elaborate.
10.	Have any administrative or judicial actions been taken?
11.	Have alternative options been identified or considered?
12.	Share a success story or lesson learnt from any negative experience in monitoring and enforcement, if any.

The background information for each country was obtained from two main sources:

- Relevant national legal measures: A list of all relevant national legal measures from the 2022 Code Status Report (5) was verified by the participants representing the eight countries in the workshop. After verification, the relevant legally-binding documents of the seven countries and the National Policy Statement of Bhutan were obtained from the UNICEF ROSA and relevant country offices. The verification process included adding in measures not mentioned in the 2022 Code Status Report, and removing measures that are not relevant. These measures were then uploaded to an online archive as part of workshop reference materials.
- A regional legal desk review that analyzed in detail the national measures of the eight countries as a result of the previous workshop held in November 2022 in Colombo, Sri Lanka (34).

### 2.3 Analysis

Deductive content analysis (17) was used to review, categorize, and analyze data collected through the workshop. A semi-structured categorization matrix was developed containing three main themes:

- Background: Background information on legal measures that give effect to the Code and general country context.
- Opportunities: Existing conditions and/or circumstances that already contribute to or can potentially be built upon or strengthened for improved or successful monitoring and enforcement.
- Bottlenecks: Existing conditions and/or circumstances that hinder or constrain successful monitoring and enforcement.

This was used as a guide for categorizing data for the following areas relevant to monitoring and enforcement:

- Background information on legal measures that give effect to the Code
- Current monitoring and enforcement actions
- Provisions in legal measures that specifically outline monitoring and enforcement mechanisms
- Provisions in legal measures that assign government agencies (or other relevant bodies) the duty or authority to carry out tasks in relation to monitoring and enforcement
- Existing mechanism or systems that can be utilized to establish or improve monitoring and enforcement
- Availability of effective monitoring tools
- Coordination among relevant government agencies.

All relevant data, as described in the Methods section, were reviewed as content and analyzed according to the three themes above (background, bottlenecks, and opportunities). Further synthesizing of the text was done for purposes of readability and understandability.

## 3 Findings

### 3.1 Afghanistan

#### 3.1.1 Background

Afghanistan adopted the Regulation on Support and Promotion of Breastfeeding (the Regulation) in 2009 with clear objectives to protect the health and safety of the child and mother, encourage and protect breastfeeding and appropriate complementary feeding, and ensure the proper use of infant feeding (and other related) products. As per the Regulation, an independent National Committee for Support and Promotion of Breastfeeding should be established to oversee monitoring and enforcement. Several government bodies are tasked with monitoring and enforcement of the Code, the Public Nutrition Department of Ministry of Public Health (MoPH) is mostly responsible in leading technical and policy level coordination, while the Environmental Health Department of MoPH and Food and Drug Administration (FDA) are tasked with supporting monitoring at import sites.

In terms of overall Code implementation, the Regulation is substantially aligned with the Code, and it also satisfies the NetCode requirement in respect of monitoring and enforcement.^5^

#### 3.1.2 Opportunities

Despite the political unrest and major shifts in policies in the country, there have still been isolated incidents of violations being reported in *ad hoc* fashion. There are also limited reports lodged for meetings and seminars that took place outside the country. There are plans underway to integrate Code monitoring into the current ongoing monitoring and supervision of public health facilities and food and drug safety and quality control programs.

There are provisions in the existing Regulation to facilitate independent monitoring and enforcement, as the Regulation provides for the establishment of a National Committee for support and promotion of breastfeeding. The Committee shall appoint professional monitors to monitor better implementation of the regulation, provide platforms for members of the public to lodge complaints, and liaise with the Attorney-General regarding prosecution. The Regulation provides for a system of registration of designated products, whereby sale of unregistered products is prohibited. This could be an important access point for monitoring and consequently enforcement, future plans for monitoring and enforcement should explore this avenue.

#### 3.1.3 Bottlenecks

Given the current political situation, past efforts and systems around monitoring and enforcement that were set up under previous administrations have been undermined or even dismantled. Currently there are no systematic monitoring and enforcement activities on the ground. There is also no channel for the public to lodge complaints regarding non-compliance. As the National Committee for Support and Promotion of Breastfeeding is not functional, there is currently no active coordination among the various relevant government bodies and no trained staff dedicated to monitoring and enforcement. There are no systems to report and track violations and no known administrative or judicial actions have been taken as there is limited capacity within government, which is further diminished by current political events. Digital marketing is reportedly growing but there is no system to monitor the marketing activities on electronic platforms. While the Regulation requires manufacturers or distributors of designated products to provide samples and information to the Ministry of Public Health in order to obtain a Certificate of Registration to be sold in the market, there has not been any action taken on registering designated products.

### 3.2 Bangladesh

#### 3.2.1 Background

Bangladesh first adopted its Breastmilk Substitutes (Regulation of marketing) Ordinance in 1984 (the Ordinance) to implement the Code. Subsequently, the 2013 Breast-milk Substitutes, Infant Foods, Commercially Manufactured Complementary Foods and the Accessories Thereto (Regulation of Marketing) Act (the Act) was adopted to close several gaps between the Code and the Ordinance. The Breast-milk Substitutes, Infant Foods, Commercially Manufactured Complementary Foods and the Accessories Thereof (Regulation of Marketing) Rules (the Rules) were adopted in 2017 to further strengthen specific provisions, including education and information. Hereinafter the Act and the Rules combined are known as “the Bangladesh Act.”

The Bangladesh Act is substantially aligned with the Code, and it also substantially satisfies the NetCode requirement in respect of monitoring and enforcement.

#### 3.2.2 Opportunities

The Bangladesh Act includes a registration requirement for products covered in the scope, which serves to ensure a certain degree of labeling compliance. Sanctions and penalties are clearly outlined, including imprisonment or fine or both with increased penalty for repeated offences. There are provisions allowing for forfeiture of goods and equipment associated with an offence and power of entry to make searches. Implementation of the Act falls under the jurisdiction of the Director of Institute of Public Health Nutrition (IPHN), receiving advice from a National Advisory Committee.

There are existing monitoring tools but changes to the tools have been proposed to improve the integration into different existing systems. There are government-sanctioned relief programs during emergencies, where some *ad hoc* monitoring is conducted.

**Figure 1:**
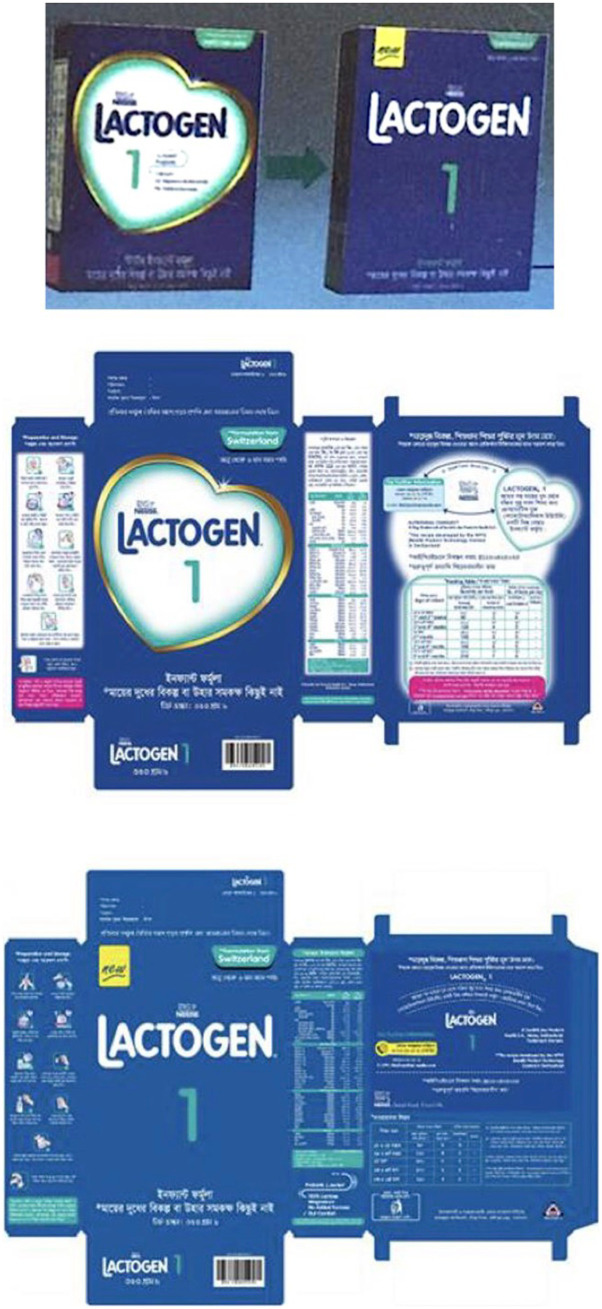
Example of plain packaging of infant formula in Bangladesh, as a result of enforcement through product registration. Idealizing graphics such as the golden heart and idealizing text on the back label have been removed.

Regarding enforcement, Bangladesh identified their product registration requirement (under IPHN) as a major enforcement point. The leverage enforcement agencies hold over companies when issuing registration licenses very likely provided one of the major avenues that contributed to major positive changes in labeling practices, resulting in plain packaging of some of the BMS products in the country (see Figure 1). Hence, enforcement can be strengthened further through more stringent customs registration and approvals procedures. In view of the existing extensive national health management information system (HMIS), there are action plans in place to collaborate with the Directorate General of Health Services (DGHS) to integrate a BMS digital monitoring application with HMIS.

#### 3.2.3 Bottlenecks

There are existing provisions in the Bangladesh Act that outline the composition and responsibilities of the National Advisory Committee to advise on overall Code implementation, but there is no mechanism to ensure monitoring is conducted free from conflicts of interest or to prevent members having a relationship with or interest in a BMS company. The public is not well informed of the Code (or its relevant national legal measures), therefore it is difficult to implement monitoring or reporting systems that engage the public. Though the existing national monitoring system is not fully functional, there are still some level of monitoring activities taking place on the ground.

Unlike the positive outcomes as seen in the plain labeling examples that resulted specifically from the comprehensive registration provisions and mechanisms, there is insufficient expertise and capacity within the enforcement agencies to expand enforcement actions to the other settings such as promotion to general public, in the health system, and on digital platforms. Hence overall monitoring on the ground has not necessarily led to enforcement actions in other areas even though there are some enforcement mechanisms in place.

### 3.3 Bhutan

#### 3.3.1 Background

The Government of Bhutan issued a Policy Statement in 2002 with the aim to promote, protect, and support breastfeeding. The Policy Statement includes a section entitled “Regulation of Marketing of Food Products and Feeding Equipment Suitable for Children below Two Years of Age,” which contains certain non-legally binding provisions relevant to the Code.

There is no legal measure to give effect to the Code in Bhutan, and due to that, existing measures do not satisfy the NetCode requirement in respect of monitoring and enforcement.

#### 3.3.2 Opportunities

Even without legal measures, food labeling and advertisements on TV and print media are monitored by the Bhutan Food and Drug Authority (BFDA) and the Bhutan InfoComm and Media Authority (BICMA) respectively. The Ministry of Health conducts periodic monitoring in health facilities across the country through the country’s implementation of the Baby-Friendly Hospital Initiative (BFHI).

Though not legally enforceable, there are provisions in the Policy Statement that require manufacturers to obtain approval from a government-approved breastfeeding committee for selling any food or feeding equipment products suitable for children below the age of two. This can be used as a stop-gap measure to monitor violations using the International Code as benchmark.

Despite not having any legal measures to give effect to the Code, Bhutan plans to incorporate Code provisions into the upcoming Food and Drug Bill. This is a good opportunity to integrate monitoring of marketing and labeling practices into the national food and drug legal regime. In the meantime, monitoring of labeling can be integrated into the existing food safety inspections conducted by the Bhutan Food and Drug Authority and during the custom duty procedure at points of entry into the country.

#### 3.3.3 Bottlenecks

Though there is some monitoring taking place on mass and print media, labeling, and at retail outlets and health facilities this is on an *ad hoc* basis when complaints are made, not in a systematic manner. The biggest bottleneck is that the Policy Statement, which is not legally-binding and therefore not enforceable, does not include provisions regarding monitoring. Hence monitoring is not integrated with any existing processes. There are no legally enforceable measures to give effect to the Code. There is also no existing monitoring tool.

### 3.4 India

#### 3.4.1 Background

India adopted the Infant Milk Substitutes Feeding Bottles, and Infant Foods (Regulation of Production, Supply and Distribution) Act in 1992. The Infant Milk Substitutes, Feeding Bottles and Infant Foods (Regulation of production, Supply and Distribution) Rules, adopted pursuant to the Act, were passed in 1993. Both the Act and the Rules went into force in 1993. Both the Act and the Rules were amended in 2003, and are to be read together (collectively known as the IMS Act).

The IMS Act is substantially aligned with the Code, and in terms of monitoring and enforcement, it substantially satisfies the NetCode requirements.

#### 3.4.2 Opportunities

Monitoring and enforcement mechanisms are clearly outlined in the IMS Act, which stipulates that the Ministry of Women and Child Development (MWCD) shall be responsible for the overall monitoring and enforcement, and written complaints of offenses may be made by authorized medical officers, food safety officers or authorized voluntary organizations, namely Association for Consumer Action for Safety and Health (ACASH), Breastfeeding Promotion Network of India (BPNI), Central Social Welfare Board (CSWB), and Indian Council for Child Welfare (ICCW).

There are provisions in the IMS Act to prohibit advertising through “electronic transmission,” which covers some forms of digital marketing, such as advertisements on social media, blog posts, and direct messages through email, text or instant messaging. Among all the voluntary organizations, BPNI is active in monitoring for violations of the IMS Act as well as the Code, in particular on the digital platforms.

The IMS Act provides sanctions, including fines and imprisonment for violations. Food inspectors and other authorized officers have powers of search and seizure when there are suspected violations of quality standards or labeling. Products may also be confiscated if they are found not compliant. This power, authorized by sections 13–19 of the IMS Act, has been exercised in the past in the state of Haryana, where products that violated the labeling provisions were confiscated. Such precedent can be leveraged for stepping up enforcement actions.

Political and government commitment to protect breastfeeding can be tapped to improve monitoring and enforcement. Monitoring and enforcement in the health systems can be strengthened through integration with the National Quality Assurance Standards process,^6^ and attempt to increase resources allocation can be made through the National Health Mission funds.

#### 3.4.3 Bottlenecks

The IMS Act is substantially aligned with the Code, however it does not include provisions that ensure independent monitoring is free from industry influence, hence it does not prohibit the Government to authorize a voluntary organization supported by a baby food manufacturer to be part of the monitoring and reporting system.

There is a lack of clarity of roles, specifically on which ministry assumes as the overseer of the IMS Act. Although the MWCD is considered to be responsible to oversee overall monitoring and enforcement of the IMS Act, the Ministry of Health is seen to be responsible for addressing violations within or related to the health system. Since there are no medical or food safety officers under MWCD, there is often ‘back and forth’ between the two ministries when it comes to acting on reported violations. As a result, enforcement has been sparse and inconsistent, and has rarely reached the investigation stage. Although a number of voluntary organizations, namely Association for Consumer Action for Safety and Health (ACASH), Breastfeeding Promotion Network of India (BPNI), Central Social Welfare Board (CSWB), and Indian Council for Child Welfare (ICCW), are tasked with making complaints of violations, only one organization, BPNI, has been conducting most of the monitoring on the ground (29). Due to lack of resources and limited capacity, the monitoring, though ongoing, is still not systematic and routinized, and has been primarily limited to focusing on digital marketing.

It was reported that there are insufficient resources allocated for monitoring and enforcement. In particular, enforcement on the ground despite having been carried out in the past, does not appear to have gained momentum.

### 3.5 Maldives

#### 3.5.1 Background

Maldives adopted the Regulation on Import, Produce, and Sale of Breast-milk Substitutes (the Regulation) under the parent law, the Law for General Regulations, to give effect to the Code in 2008. The Maldives Regulation is substantially aligned with the Code, and fully satisfies the NetCode requirement in respect of monitoring and enforcement.

#### 3.5.2 Opportunities

The Regulation includes provisions that clearly outline monitoring and enforcement mechanisms. The provisions authorize the Maldives Food and Drug Authority (MFDA) to be responsible for overall implementation, monitoring and enforcement; and empower members of the public to lodge complaints of violations. The powers and responsibilities of the National Advisory Board, which consists of various government agencies, as well as nongovernmental organization representatives and members of the public, are outlined. Safeguards against conflicts of interest within the advisory board and monitoring and enforcement mechanisms are in place. Registration of products is required, which has proved to be a very effective mechanism for monitoring and potentially effective in enforcement if carried out properly. Sanctions and penalties are clearly outlined.

Despite being limited to the central level, monitoring is systematically conducted by the MFDA through biannual market surveillance. In addition, the MFDA has established a monitoring tool and mechanism for receiving reports of violations via a hotline and email.

The country reported strong internal coordination and collaboration within the government. For instance, systematic and effective monitoring is conducted at four specific ports of entry (two at national level and two at subnational levels) due to the collaboration and coordination between MDFA and the Maldives Customs Service (MCS). The National Advisory Board is active in monitoring and providing guidance on Code implementation in the country.

It is within the MFDA’s power to issue cease and desist orders and written warnings to remove products that are not compliant with the Regulation. The MDFA can also revoke the certificates of registration of products and professional licenses, and together with the MCS, licenses for sales and import. These are useful tools to compel compliance by companies and health professionals alike.

Maldives has a comprehensive Integrated National Nutrition Strategic Plan (INNSP) that addresses protection of breastfeeding and optimal complementary feeding as key priorities, but enforcing national regulation of marketing of breastmilk substitutes was only briefly mentioned in the plan (31). Integration of monitoring and enforcement into the INNSP can be a way to ensure such activities are institutionalized as a key part of the national nutrition action plan, are allocated with the necessary resources, and properly monitored and evaluated.

#### 3.5.3 Bottlenecks

Although monitoring is being conducted by the MFDA and the MCS, with the exception of customs at the ports of entry, monitoring is mostly limited to the central level. Monitoring and enforcement activities only started to take form some 10 years after the Regulation was adopted and progress was reported to be slow.

Monitoring of digital platforms, where both registered and unregistered BMS products are marketed, is lagging severely amid the rampant promotion. There is almost no monitoring conducted at subnational levels, which leaves promotion at sites such as health facilities, pharmacies, and retail outlets, as well as other promotion activities outside the capital area unchecked. There is generally a lack of resources allocated for monitoring and enforcement, and very little awareness on the Regulation within the government. Capacity to support monitoring from civil society is limited, and members of civil society do not have a clear idea about who the key stakeholders are to liaise with regarding monitoring and supporting enforcement, and for advocacy purposes.

The inability to impose sanctions on the violators remains a major bottleneck. The Regulation was adopted under the Law for General Regulations, which does not provide the authority to impose sanctions. Hence the violations cannot be sanctioned under the Regulation even though it originally included fines as a form of sanction. The section that outlines sanctions for violations has been removed, and will only be enforceable until the impending Food Security Bill is enacted as the Regulation’s parent bill (expected to be in May 2024).

### 3.6 Nepal

#### 3.6.1 Background

Nepal adopted the Breastmilk Substitutes (Marketing Control) Act in 1992, and in 1994, the Breastmilk Substitutes (Marketing Control) Regulation 1994 was passed to implement the parent Act, by setting out the required procedures for labeling approval, and monitoring and inspection (both combined, known as the Act). The Act is currently under review, a revised Act has been drafted with the support from a task force consisting of lawyers, health officials, UNICEF, and WHO staff. The revised Act has been submitted to the Ministry Law, Justice and Parliamentary Affairs for approval pending tabling in Parliament.

While the Act is only moderately aligned with the Code, its existing monitoring and enforcement provisions substantially satisfy the NetCode requirements.

#### 3.6.2 Opportunities

The Act has clearly identified monitoring and enforcement mechanisms, including designating the Ministry of Health with the authority and responsibility to carry out and oversee monitoring of compliance. The Ministry also chairs the multi-disciplinary Committee for the Promotion and Protection of Breastfeeding to advise on investigation or initiation of cases against violators. On the recommendation of the Committee, the Ministry of Health is given the authority to appoint inspectors to monitor compliance of companies, health facilities, and health workers. An inspector may take action instructed by the Committee, including filing a case in court with the assistance of a government lawyer upon completion of investigation. There are provisions in the Act outlining product certification and labeling approval requirements, which provide the additional platform for effective built-in mechanisms for monitoring and enforcement. Sanctions for violations have been identified in the Act, including suspension and revocation licenses, permits or authority, fines or imprisonment, and the owners, partners or CEOs of corporate bodies concerned are liable to these punishments.

There is regular and systematic monitoring conducted on the ground, as well as in emergencies as they arise. Monitoring activities are integrated into existing regulatory systems including routine food inspections and there is a web-based reporting system set up within the Multi-Sector Nutrition Programme (MSNP). Monitoring and reporting of violations can also be included as part of the Baby-Friendly Hospital Initiative (BFHI) assessment, the Nepal Nutrition and Food Security (NNFS) Portal web-based reporting system, and customs and border control. Suggestions to integrate monitoring findings with existing food inspection agencies under the Department of Food Technology and Quality Control (DFTQC) and Department of Industry, Commerce and Supplies (DICS) were made.

The impending amendment of the Act that aims to align the existing legal measures with the Code provides an opportunity to strengthen the areas in monitoring and enforcement that have fallen short, in particular identifying focal points and training of staff.

#### 3.6.3 Bottlenecks

Although the Act has been in place since 1992, it is reported to be ineffective due to lack of identification of focal points for monitoring and enforcement at the central level as well as district levels. Another major bottleneck is the overall lack of trained officials designated to oversee monitoring and the lack of coordination between government agencies tasked with monitoring and enforcement. As a result, though monitoring takes place on the ground, enforcement is lacking.

Marketing on social media platforms is becoming the dominant form of promotion of BMS and related products. However, there has not been systematic monitoring and enforcement to address digital marketing.

There are a number of existing monitoring tools for different settings, but the tools are not user-friendly, which acts as a barrier to rapid reporting. Since the Act was adopted in 1992 and the Regulation in 1994, the scope is narrower than the scope of the Code as clarified in subsequent World Health Assembly Resolutions. Even if enforcement was in place, many of the products being marketed inappropriately would not have been captured. The lack of safeguards in the Act to prevent the appointment of individuals with ties to companies to the Committee makes it impossible to prevent conflicts of interest from arising. There has been industry influence reaching health workers, which makes monitoring and enforcement in health facilities challenging.

### 3.7 Pakistan

#### 3.7.1 Background

Pakistan adopted the Protection of Breast-feeding and Child Nutrition Ordinance in 2002. In 2010, the 18th constitutional amendment transferred many federal level responsibilities to provincial governments in a major restructure of Pakistan’s political and legal systems (32). Even though the federal Ordinance devolved to the provincial level for implementation, this analysis uses the federal Ordinance as the basis instrument/foundation for discussion as the responsibility of the federal government still remains in areas that are crucial to Code implementation such as public health policy norms and information, and trade and interprovincial regulation and coordination. Also, the provincial laws are in *pari materia* (on the same subject or matter) with the federal Ordinance, only with necessary changes to reflect the transfer of functions and responsibilities to provincial authorities.

In 2009, the Protection of Breast-feeding Rules were adopted to implement the Ordinance covering among others, the constitution of the National Infant Feeding Board, its powers and functions. The Rules also set out the required information for information and educational materials targeting health professionals, and labeling restrictions. The 2002 Ordinance and 2009 Rules are to be read together (the federal Ordinance). Both were adopted as federal measures that extend to the entire country along with the provincial BMS Acts.

Overall, the federal Ordinance is moderately aligned with the Code, and only marginally satisfies the NetCode requirement in respect of monitoring and enforcement.

#### 3.7.2 Opportunities

The Ministry of National Health Services, Regulation & Coordination (MoNHSR&C) and Provincial Department of Health have taken actions to ensure there is some level of monitoring and enforcement at the federal and provincial levels. For instance, monitoring is active in the health system, and warning letters were issued during the COVID pandemic and the subsequent flood emergency in 2022 by the MoNHSR&C in response to reported violations. In a few provinces including Punjab, through the provincial Infant Feeding Board, food regulatory authorities are also assigned with the responsibilities of monitoring and enforcement.

The federal Ordinance provides the Infant Feeding Board authority to call for investigations and the federal government may delegate its authority to the concerned provincial government where the complaint is filed. The federal Ordinance and the provincial Acts provide for the designation of qualified persons as inspectors to conduct inspection, investigation, and prosecution upon recommendation from the Infant Feeding Board.

Sanctions, confiscation of products from convicted manufacturers, suspension of medical license, and other penalties (after trial) are clearly stated in the federal Ordinance. With the significant role of the Infant Feeding Board in monitoring and enforcement, one major accomplishment has been the removal of industry representatives from the Infant Feeding Board. There is avenue for public enforcement whereby any person is entitled to file a complaint concerning a violation of the 2002 Ordinance or 2009 Rules to the National Infant Feeding Board. It was also reported that digital marketing is being monitored by civil society intermittently. Plans have been made to include monitoring and enforcement into existing systems, such as the Multisectoral Nutrition Monitoring Mechanism, District Health Information System, and the Food Safety Authority, and customs and border control.

#### 3.7.3 Bottlenecks

Although the Infant Feeding Board, which is chaired by the Ministry of Health, or a Provincial Committee, has the authority to call for investigations when reports of violations are made, monitoring has not been conducted in a systematic manner. And while there is still some monitoring on the ground, most monitoring and enforcement actions are concentrated in the health system, and monitoring and enforcement are weak in other areas such as retail and promotion to general public (advertisement). There is no specific monitoring tool or framework to facilitate systematic monitoring. There is also a lack of inter-agency coordination, and limited capacity and resources at the federal and provincial levels to carry out monitoring and enforcement activities. Although there is some monitoring conducted on digital marketing by civil society, it is not systematic and extensive, but plans have been made to strengthen monitoring on the digital platforms.

Even though suggestions were made to mobilize agencies such as the Punjab Food Authority (PFA), Drug Regulatory Authority of Pakistan (DRAP), and Provincial/District Disaster Management Authority (PDMA/DDMA) to carry out enforcement, and for civil society organizations to participate in monitoring, the lack of resources seem to have hindered the plans, and so far only the PFA has been involved in some monitoring and enforcement.

Political instability, together with the devolution of the Federal Ordinance to the provincial level for implementation through provincial laws, have created challenges in providing government officials capacity building activities to strengthen enforcement.

### 3.8 Sri Lanka

#### 3.8.1 Background

Sri Lanka was one of the first countries to implement the Code following its adoption in 1981. The Sri Lanka Code for the Promotion, Protection and Support of Breastfeeding and Marketing of Designated Products was adopted in 1983 and amended in 2002. The Sri Lanka Code is currently under review, relevant government agencies are receiving technical consultation as a new Act is being drafted.

The Sri Lanka Code designates a number of Ministries, including the Ministry of Health, Trade, Food and Marketing, Justice, and Labour, to be responsible for Code implementation, and designates the Ministry in-Charge to appoint a Monitoring Committee to oversee the function of monitoring. However, it is unclear which Ministry is primarily responsible as the Sri Lanka Code was adopted under the Consumer Affairs Authority Act, which is generally administered by the Ministry of Trade. However, in practice, the Ministry of Health, specifically the Family Health Bureau within it, has been handling the administration of the Sri Lanka Code.

The Sri Lanka Code is moderately aligned with the Code, and only partially satisfies the NetCode requirement in respect of monitoring and enforcement.

#### 3.8.2 Opportunities

Despite the various challenges that restrict monitoring and enforcement, there is still monitoring taking place, with much of it in the health systems due to the health workers’ and Ministry of Health staff’s awareness on the Code. This indicates there is existing capacity for the Ministry of Health to oversee monitoring, should the mechanisms for monitoring be established or be identified with the impending Act that is being drafted. As such, Ministry of Health participants at the Kathmandu Workshop have outlined the elements to be included in a monitoring tool, modifying from the NetCode universal form. There are also plans to conduct a NetCode periodic monitoring exercise in 2024, as a follow-up to the Net Code monitoring in 2019.

It is also reported that there is ongoing advocacy supported by the Ministry of Health to sensitize other government departments on the importance of Code monitoring. A notable achievement is that administrative actions such as Ministry of Health sending warning letters to violators, including professional associations and health professionals, have been taking place.

Out of necessity (see “Bottlenecks” for more context), the country is now in the process of drafting a new comprehensive law for the protection of breastfeeding. The opportunity to draft a brand-new Act housed under the Ministry of Health that provides relevant agencies with appropriate powers and authorities to carry out and oversee monitoring and enforcement and proportionate sanctions and penalties will greatly improve the current monitoring and enforcement situations.

#### 3.8.3 Bottlenecks

Although the Ministry of Health has been implementing the Code in practice, it was adopted under the Consumer Protection Act, administered by the Ministry of Trade. The Ministry of Health has no enforcement powers until the Code is adopted as a legal measure under the jurisdiction of the Ministry of Health, with powers of monitoring and enforcement properly conferred. As a result, there has been no enforcement on the Sri Lanka Code, which is a major bottleneck.

There is also lack of clarity on the composition of the Monitoring Committee and the extent of jurisdiction regarding investigation and prosecution. There is no existing monitoring tool and no requirement on such mechanisms being free from commercial interest. There are also no sanctions or penalties outlined.

Due to the existing conditions of Code implementation, national funding and resources allocated for monitoring and capacity building are severely lacking. Ministry of Health has not been able to effectively monitor digital marketing that has become rampant, as the definition of advertising in the Sri Lanka Code is too narrow to cover digital platforms.

### 3.9 Key themes across countries

#### 3.9.1 Bottlenecks

Across the eight countries, even though there have been varying levels of monitoring activities taking place at the community level, they are generally intermittent and *ad hoc*. Systematic and ongoing monitoring is still insufficient. In particular, monitoring is not well-integrated into relevant enforcement mechanisms, and monitoring findings, if any, are not efficiently ‘fed’ to the designated enforcement agencies. There is also lack of coordination and collaboration among key agencies regarding enforcement. Countries reported that sanctions and penalties are inadequately imposed. In some cases, there is a lack of a clearly identified legal structure and adjudication system upon which enforcement systems rely, and lack of functional administrative or enforcement mechanisms that culminate in sanctions. Overall, sanctions and penalties are found to be insufficient to deter violations. Most countries reported uncertainty regarding enforcement in the context of digital marketing.

#### 3.9.2 Opportunities

Product registration or licensing has found to be relatively effective entry points for monitoring and enforcement. For example, registration requirement is included in the Bangladesh Act and has proved to be useful to ensure compliance. In most countries, authority for designated agencies to carry out monitoring and enforcement actions and sanctions are provided in the legal measures. Also, across countries, civil society organizations have found to be strong partners in providing support and expertise around monitoring.

## 4 Discussion

### 4.1 Overview on national monitoring and enforcement mechanisms

The findings above provided a descriptive analysis of the bottlenecks and opportunities of the monitoring and enforcement of Code-based legal measures of Afghanistan, Bangladesh, Bhutan, India, Maldives, Nepal, Pakistan, and Sri Lanka. Except for Bhutan which does not have any legal measures that give effect to the Code, all countries in the region have, to varying degrees, provisions that provide for monitoring and enforcement actions and mechanisms, but some are more comprehensive and clearer (e.g., India, Maldives, and Bangladesh are substantially and fully aligned with the NetCode requirements regarding monitoring and enforcement) than others (e.g., Pakistan and Sri Lanka only marginally and partially satisfy the NetCode monitoring and enforcement requirements).

#### 4.1.1 Common bottlenecks

Key bottlenecks reported by countries are discussed in detail in sections below, including digital marketing, lack of integration between monitoring and enforcement, insufficient collaboration and coordination, and inadequate sanctions.

##### 4.1.1.1 Digital marketing juggernaut

With strategies like cross-border marketing and user-generated content, most countries reported being unsure on how to tackle digital marketing regarding enforcement. Both India and Pakistan reported civil society is active in monitoring digital platforms. In India, the IMS Act includes provisions restricting promotion through “electronic transmission,” which is considered a subset of digital marketing that focuses on the internet instead of all digital platforms. Such ‘e-marketing’ typically focuses on social media, blogs, as well as direct communications through emails and online messengers, allowing for interactive communications with their target audience rather than those that are static and not targeted. However, due to bottlenecks in enforcement and lack of sanctions and penalties, the monitoring alone seems to have little effect in deterring the rampant digital marketing (18).

Monitoring of digital marketing is often perceived as taxing as it is a newer form of marketing that has gone ‘viral’ in the last two decades, involving large amounts of materials that need to be collected, reviewed, and analyzed. Governments reported feeling unsure as to how to tackle the previously unaddressed areas, including targeted ads appearing on pregnant mothers’ search engine or phone apps, social media groups, influencer or user-generated content, and cross-border marketing. The Code prohibits all forms of promotion, and many marketing activities found on digital platforms are thus included in the Code and relevant national measures. Although certain practices do call for greater regulatory clarity and specificity, and in some cases additional laws or regulations may be needed, promotion on the internet is subject to the same rules as conventional promotion generally. The unfamiliarity in this area has incapacitated enforcers even where provisions in legal measures are adequate.

##### 4.1.1.2 Monitoring findings do not feed into the system to enable enforcement

Even though monitoring exists to varying degrees for all countries, countries tend to wait for new updated laws to start ‘ramping up’ or focusing on enforcement. In some cases, monitoring has been conducted by larger international non-governmental organizations (NGOs) (30), but the system or protocol does not seem to trickle down to governments, and there are capacity and resource issues that make it unsustainable for local civil society organizations to implement consistent and systematic monitoring. Country participants have provided concrete examples where existing registration (customs department) or inspection (food inspection at retail sites) mechanisms can be used to enforce Code-related measures. However, more often than not, the monitoring system(s) (usually conducted by civil society or Ministry of Health) is not connected or integrated with the existing enforcement mechanisms, which means monitoring findings do not feed into the system to enable enforcement.

##### 4.1.1.3 No systematic, routinized, and ongoing monitoring

Most countries reported having some form of monitoring on the ground, either through civil society, or designated government authority. However, the monitoring is either not systematic, or not sustainable enough for it to be a routine, ongoing activity. Countries like Bangladesh, Bhutan, and Maldives reported the monitoring system as weak, not “institutionalized,” and needing to be expanded and strengthened. Due to lack of resources, monitoring in India is not systematic. Monitoring is both ongoing and systematic in Maldives, but it only covers the central level, leaving promotion at sites such as health facilities, pharmacies, and retail outlets, as well as other promotion activities outside the capital area unchecked. While monitoring of labeling (including imported products at customs) and digital marketing can be done at the national level, monitoring of health facilities, retail outlets, and other sites and platforms need to be conducted beyond the national level as marketing practices vary from place to place. For Nepal, due to insufficient training of monitoring and enforcement staff, even though monitoring is regular (periodic) and systematic, it is reported as not effective enough to lead to enforcement.

##### 4.1.1.4 Inadequate or lack of appropriate sanctions

Even though most of the national legal measures include provisions identifying sanctions that should be imposed in the case of violations, they are not severe enough to deter violations. There is a lack of range of appropriate penalties that should include a variety of criminal as well as administrative sanctions. In some countries, the legal structure and adjudication system are not clearly identified, which contributes to non-functional or inefficient administrative or enforcement systems, making it difficult to culminate in sanctions.

##### 4.1.1.5 Poor coordination among designated agencies

Though countries can generally identify existing enforcement mechanisms with which monitoring can be integrated, often a lack of collaboration and coordination (e.g., Afghanistan and Pakistan), and absence of clear designation of roles and responsibilities in the national legislation (e.g., Sri Lanka) contribute to inadequate or no enforcement. The overall administration, monitoring, and enforcement is usually overseen by the Health Ministry, which is usually tasked with many responsibilities, including setting up a product registry to facilitate monitoring and inspection, appointing an advisory board of technical specialists to carry out special functions such as vetting of information materials, reviewing of monitoring reports, and advising on breastfeeding promotion. Often, these tasks are not well coordinated and collaborated with other government agencies who may have the sole or lead jurisdiction to implement, monitor and enforce advertising or marketing laws. Even for countries that have monitoring mechanisms established, poor inter-agency coordination of roles and responsibilities is a barrier to an integrated monitoring and enforcement system.

##### 4.1.1.6 Lack of financial and human resources

Funding for Code monitoring and enforcement activities are usually not included in national budgets and human resource plans. When legal measures and the relevant activities identified in them are not tied to specific budgetary systems a country uses to allocate resources for Code-related government obligations, it is difficult for such activities to secure government commitment to become routinized and institutionalized.

#### 4.1.2 Common opportunities and effective strategies

Key opportunities and effective strategies reported by countries are discussed below, including utilizing product registration, provisions addressing authority to monitor (and enforce) and sanctions in national legal measures, and expertise of civil society to strengthen monitoring and support enforcement.

##### 4.1.2.1 Product registration as entry point

The product registration requirement included in the Bangladesh Act has proved to be effective to ensure compliance with quality and labeling provisions. It was instrumental in achieving plain packaging of some BMS products in the country. Recent examples of plain labeling of BMS products were shown during the Kathmandu Workshop. Compared to the same products (from the same brand and company) marketed in other countries in the region, the labeling of the products shown from Bangladesh did not have any idealizing images or text, contained no nutrition claims, and no statement that suggests the product is comparable to breastmilk.

##### 4.1.2.2 Authority to monitor and sanctions

Most of the countries in the region have provisions in their legal measures to provide authority for designated agencies to carry out monitoring and enforcement actions, as well as provisions addressing sanctions and penalties. Even though many of these provisions need further clarification, they provide ready and existing basis to prompt some enforcement actions. Similarly, even though sanctions may not be appropriate and sufficient, the provisions that address sanctions can be harnessed to ensure oversight and correction.

##### 4.1.2.3 Civil society as partners on strengthening knowledge and capacity on monitoring and advocacy

There has always been monitoring activities at the community level in the South Asia region, often conducted by civil society, such as the International Baby Foods Action Network and its national groups, Alive & Thrive, and local civil society. Many of them have extensive knowledge on the Code, and work closely with the country’s government. Using India as an example, governments can tap into the human resources, knowledge, and social capital and include them as partners in monitoring, including establishing or strengthening systems to integrate monitoring with enforcement and developing monitoring tools. Broad support from the public and strong political will at the top are crucial to protect a law and its enforcement from being ignored. Civil society can also be mobilized to support advocacy efforts at both levels, and to call out harmful company practices and conduct.

### 4.2 Recommended actions

Based on the bottlenecks and opportunities reported from the eight countries, actions below are recommended with the aim to strengthen existing monitoring and enforcement situations.

#### 4.2.1 Monitoring and enforcement activities as institutionalized activities in national budgets

Ensuring adequate financing for monitoring and enforcement should be described as an investment in health and not a cost. When monitoring and enforcement are included as routinized activities in the national budget, they are more likely to secure government commitment and become ‘institutionalized’. It is important to have well-costed plans to inform adequate allocations of resources. Well-functioning enforcement systems can generate revenue from fines and other financial sanctions that can be used to fund the implementation of legal measures as well as other relevant government initiatives.

#### 4.2.2 Including appropriate range of (administrative and criminal) sanctions as deterrents

Legal measures must include a system of sanctions that can be imposed when violators are found guilty. Penalties imposed must be appropriate and heavy enough to act as deterrents, such as fines based on the size of violators and frequency of violations. Apart from criminal sanctions normally imposed by a court of law (i.e., fines and imprisonment), other effective administrative sanctions such as warnings, corrective action notices, confiscation of goods (useful for violations relating to labeling or quality of the product), suspension or revocation of a license to manufacture, import, or sell a product should also be considered.

#### 4.2.3 Using registration and license as key enforcement and sanction tools

Even for countries with legal measures, often there is a lack of clear monitoring and enforcement mechanisms identified, no authority designated to relevant enforcement agencies, or the absence of a sound legal infrastructure. The issuance of company and product registration licenses determines whether a company can operate in a country and whether a product can enter the market to be sold. This gives enforcement agencies leverage over companies regarding compliance. A government can determine that companies and designated products need to be registered and approval needs to be sought for company operation and products to be imported, manufactured or sold. Where there is non-compliance with the national measures, licenses can be withheld or revoked as penalties. Using company operation licenses in enforcement and as sanctions can be especially useful when addressing digital marketing, where many marketing activities are not always directly or explicitly linked to the products.

#### 4.2.4 Reviewing or adopting new legal measures

Many countries in the region are reviewing their existing law. When drafting or revising a law, monitoring and enforcement systems, as well as sanctions, need to be addressed and included from the outset. There need to be provisions that clearly give authority (and responsibility) to appropriate government agencies to carry out enforcement actions, at the national and sub-national levels where necessary. Sanctions and penalties must be proportionate enough to deter the industry from violating the law.

#### 4.2.5 Harnessing health system as key point for monitoring and enforcement and to prevent conflicts of interest

Apart from and beyond national legal measures, health facilities and other bodies in the health system can establish best practices that incorporate Code monitoring and integrate with enforcement mechanisms (e.g., BFHI). However, due to their professional influence over mothers and parents, health workers and their associations can be regular entry points and prime targets of the baby food industry’s marketing strategy. Hence, implementing safeguards against conflicts of interest is important in preventing companies from using health professionals and the health system as a conduit to promote to mothers.

#### 4.2.6 Tackling monitoring of digital marketing

Although certain gray areas exist that make enforcement of digital marketing regulations challenging, mainstream promotional practices that appear on digital platforms are not exempt from the Code or national measures. Many of these practices can be subject to enforcement, and certainly can be monitored. The newly launched 2023 WHO Guidance can provide more practical support in this area. New technologies, such as artificial intelligence, can also be used to auto-detect and analyze violations on digital platforms, reducing demands on human resources to manually collect violations.

#### 4.2.7 Building regional knowledge-sharing platforms

The regional activities on the Code have facilitated proactive communications and coordination between public health and legal officials and advocates to ensure the national measures adequately capture marketing practices that undermine health. Building or sustaining a regional knowledge-sharing network can provide a platform for collaboration on developing tools, designing monitoring systems, and exchanging enforcement success stories.

### 4.3 Strengths and limitations of study

#### 4.3.1 Strengths

This study fills the gap found in Code-related studies, most of which that address monitoring and enforcement tend to focus on assessing the relevant legal provisions, but not how effective the national monitoring or enforcement system is, and the extent of monitoring and enforcement that take place on the ground. There is generally a lack of analysis around whether monitoring and enforcement mechanisms are in place. Its workshop-as-research approach (16) also allows deep analysis that includes great details and nuances embedded in dialogs and interactions among workshop participants about situations on monitoring and enforcement, which are otherwise not provided by just analyzing the legal measures or data from surveys or interviews.

#### 4.3.2 Limitations

A limitation of the workshop-as-research approach is that participants’ responses may be influenced by their organizational affiliations and professional mission which could contribute to biases. Participants may only have partial information that is not representative of the entire situation, as some monitoring and enforcement activities take place at sub-national levels. Monitoring and enforcement are context-sensitive and dependent, and ever changing. Findings from this study may not be applicable in other specific national contexts. This study is grounded in content analysis of data, without *a priori* framework. Hence it may be difficult to replicate. Due to the focus and nature of the study, it was also not designed to provide specific reports of Code violations or country-specific status of compliance. This study focuses on identifying the opportunities and bottlenecks of monitoring and enforcement. Even though the opportunities include some cases and analysis of effective strategies, the focus is to help countries identify and capitalize on their existing opportunities to improve monitoring and enforcement, therefore it does not provide in-depth case studies of how countries have successfully navigated obstacles associated with monitoring and enforcement. However, such case studies would be an important gap to address in future studies to help inform policy and regulatory improvements. Nonetheless, the authors believe the overarching themes in bottlenecks and opportunities do provide some common ground insight for countries to tackle challenges in monitoring and enforcement of the Code.

## 5 Conclusion

### 5.1 Delay no more on enforcement

The 1981 Code calls upon governments to ‘translate’ the Code into enforceable laws at the national level. The 2005 Innocenti Declaration On Infant and Young Child Feeding urges governments to establish sustainable enforcement mechanisms and WHA Resolution 61.20 in 2008 calls on governments to scale up monitoring and enforcement efforts while keeping in mind resolutions to avoid conflicts of interests. The NetCode toolkit has been established to assist governments in setting up appropriate, sustainable, and effective monitoring and enforcement mechanisms. New technologies to aid monitoring and enforcement are becoming more accessible, and the new 2023 WHO Guidance also provides a path forward to guide governments on tackling digital marketing. However, the findings show that even though seven out of eight countries in the region have laws and other regulatory measures in place, there is little enforcement action, albeit each country has some level of monitoring. Sanctions and penalties are inappropriate and inadequate to deter violations and are mostly not imposed properly. Monitoring and enforcement are not included as routinized activities in the national budgets. Identifying the common bottlenecks also means there are existing or potential opportunities for actions to dismantle such barriers. The plain packaging of BMS products in Bangladesh achieved through leveraging product registration and license has provided other countries with a tangible and ‘do-able’ example. Though there are some gaps in national legal measures, monitoring and enforcement should not wait until all gaps are filled to start. To give effect to existing legal measures, governments, as duty bearers to defend human rights and enforce national laws that protect maternal and child health, must galvanize the will and leadership to mobilize monitoring and enforcement and put in place functional mechanisms that can culminate in sanctions severe enough to deter violations. Delaying any further runs the risk of disregarding the courageous stand nations in South Asia have taken as early law adopters to give effect to the Code to hold companies’ erroneous conduct to account.

To date, very few countries have set up good monitoring and enforcement systems that give effect to their national Code-based measures. However, when monitors and enforcers show that they understand Code issues and stand firm about giving effect to their legal measures, in some cases, companies do take action to comply with the law. It is often a step by step process, but every little step taken will make a difference to the way companies view Code compliance.

## Data Availability

The original contributions presented in the study are included in the article/supplementary material, further inquiries can be directed to the corresponding authors.

## Footnotes

^1^Along with the 2016 WHO Guidance on ending the inappropriate promotion of foods for infants and young children (‘the 2016 WHO Guidance’) that was included in the WHA resolution 69.9 [2016].

^2^Hereinafter they will be broadly referred to as ‘BMS and related products’.

^3^These are the eight countries represented by the UNICEF Regional Office for South Asia (UNICEF ROSA): Afghanistan, Bangladesh, Bhutan, India, Maldives, Nepal, Pakistan, and Sri Lanka.

^4^Countries with WHO Member State status.

^5^The assessment of the Regulation provisions does not reflect monitoring and enforcement situations on the ground, especially in view of the political unrest and major shifts in policies in the country.

^6^Launched in 2013 with an aim to improve the Quality of Care in public health facilities of India.
